# Semiautomated surveillance of deep surgical site infections after colorectal surgeries: A multicenter external validation of two surveillance algorithms – ERRATUM

**DOI:** 10.1017/ice.2022.213

**Published:** 2023-07

**Authors:** Janneke D.M. Verberk, Tjallie I.I. van der Kooi, David J. Hetem, Nicolette E.W.M. Oostdam, Mieke Noordergraaf, Sabine C. de Greeff, Marc J.M. Bonten, Maaike S.M. van Mourik

In the above article^1^, table 2 was incorrect; below is the corrected table:

**Table 2:** Algorithm performance (% (95%-confidence interval), unless specified other)

	Classification model					Regression model					Modified classification model				
	Sens	Spec	PPV	NPV	% red.	Sens	Spec	PPV	NPV	% red.	Sens	Spec	PPV	NPV	% red.
**Hospital A**	100 (59.0-100.0)	90.4 (85.4-94.1)	26.9 (11.6-47.8)	100 (97.9-100.0)	87.4	100 (39.8-100.0)	78.1 (71.4-83.9)	9.0 (2.5-21.8)	100 (97.5-100.0)	76.5	100 (59.0-100.0)	77.8 (71.3-83.4)	13.7 (5.7-26.3)	100 (97.6-100.0)	75.2
**Hospital B**	100 (29.2-100.0)	89.3 (82.3-100.0)	18.8 (4.0-45.6)	100 (96.6-100.0)	87.2	100 (29.2-100.0)	78.9 (70.1-85.9)	11.1 (2.3-29.2)	100 (95.9-100.0)	76.8	100 (29.2-100.0)	80.1 (71.9-86.9)	11.1 (2.3-29.2)	100 (96.3-100.0)	78.3
**Hospital C**	85.7 (42.1-99.6)	92.2 (87.6-95.5)	27.3 (10.7-50.2)	99.5 (97.1-99.9)	89.7	100 (59.0-100.0)	80.0 (73.8-85.3)	14.9 (6.2-28.3)	100 (97.7-100.0)	77.3	100 (59.0-100.0)	77.6 (71.2-83.1)	13.2 (5.4-25.3)	100 (97.7-100.0)	75.0
**Hospital D**	72.7 (39.0-93.9)	97.5 (92.9-99.5)	72.7 (39.0-93.9)	97.5 (92.9-99.5)	91.6	NA	NA	NA	NA	NA	100 (71.5-100.0)	89.2 (82.2-94.1)	45.8 (25.6-67.2)	100 (96.6-100.0)	81.7

Abbreviations: Sens=sensitivity; Spec=specificity; PPV=Positive predictive value; NPV= negative predictive value; % red=percentage of workload reduction in number of medical records to review.
